# Supplementary material for: Development of a Fast Liquid Chromatography Coupled to Mass Spectrometry Method (LC-MS/MS) to Determine Fourteen Lipophilic Shellfish Toxins Based on Fused–Core Technology: In-House Validation
Source: Mar Drugs. 2021 Oct 24;19(11):603. doi: 10.3390/md19110603 (PMC8622501; doi:10.3390/md19110603)
Supplement: Supplementary file 1 [file marinedrugs-19-00603-s001.zip › marinedrugs-1411341-supplementary.pdf]

Supplementary material

Development of a fast liquid chromatography coupled to mass spectrometry method (LC-MS/MS) to determine fourteen lipophilic shellfish toxins based on fused–core technology: In-house validation

Table S1. Precision results for the analysis of naturally contaminated samples

Sample	Matrix	Toxin	RSDr % (*n* = 2)	RSDR % (*n* = 5)
502	Mussel	Total OA	< LOQ	< LOQ
504	Mussel	Total OA	*	7.9
505	Mussel	Total OA	*	6.90
506	Mussel	Total OA	*	7.3
507	Mussel	Total OA	*	7.0
514	Mussel	Total OA	2.7	9.7
515	Mussel	Total OA	9.0	11.6
516	Mussel	Total OA	1.7	12.1
517	Mussel	Total OA	7.0	10.8
518	Mussel	Total OA	6.1	13.2
519	Mussel	Total OA	3.1	12.6
520	Mussel	Total OA	5.8	< LOQ
521	Mussel	Total OA	4.6	14.4
522	Mussel	Total OA	2.4	< LOQ
639	Cockle	Total OA	1.1	22.3

646	Mussel	Total OA	3.9	19.5
660	Mussel	Total OA	1.4	17.2
688	Mussel	Total OA	1.6	8.4
696	Mussel	Total OA	3.4	8.7
698	Mussel	Total OA	3.1	8.4
717	Cockle	Total OA	3.4	14.8
1536	Cockle	Total OA	3.2	9.6 **

* Data missing

** $n = 7$
